# Association of gut microbiota with overweight/obesity combined with gestational diabetes mellitus

**DOI:** 10.1099/jmm.0.002010

**Published:** 2025-05-14

**Authors:** Shanshan Mei, Yisheng Chen, Yan Long, Xueqing Cen, Xueqin Zhao, Xiaoyan Zhang, Jingyi Ye, Xiaoli Gao, Chunyan Zhu

**Affiliations:** 1 Department of Obstetrics, Guangzhou Women and Children’s Medical Center, Guangzhou Medical University, Guangzhou, 510623, PR China; 2 Department of Epidemiology and Health Statistics, School of Public Health, Guangzhou Medical University, Guangzhou, 511436, PR China; 3 Department of Laboratory, Guangzhou Women and Children’s Medical Center, Guangzhou Medical University, Guangzhou, 510623, PR China; 4 Faculty of Dentistry, National University of Singapore, Singapore, Singapore; 5 Saw Swee Hock School of Public Health, National University of Singapore, Singapore, Singapore

**Keywords:** gestational diabetes mellitus, gut microbiota, obesity, overweight

## Abstract

**Introduction.** Gestational diabetes mellitus (GDM) is one of the most common complications of pregnancy and negatively affects the health of mothers and infants. The aim of this study was to explore the associations between gut microbiota and the risk of GDM amongst overweight/obese women, and the interaction between gut microbiota dysbiosis and overweight/obesity in pregnant women with GDM.

**Hypothesis/Gap statement.** Previous studies revealed that there may be a link between gut microbiota and GDM and obesity, but these studies have not reported the associations between gut microbiota and the risk of GDM amongst overweight/obese women, whilst the interaction between gut microbiota dysbiosis and overweight/obesity in pregnant women with GDM remains unknown.

**Aim.** Based on a prospective cohort study, we explored the composition of gut microbiota in overweight/obese pregnant women and its association with GDM.

**Methodology.** Participants (n=1820) were enrolled from the Pregnancy Metabolic Disease and Adverse Pregnancy Outcome cohort in Guangzhou, China, between 2019 and 2021. The participants’ information and faecal samples were collected, and the relative abundance of faecal microbiota was profiled using 16S rRNA V4 region sequencing. Pregnant women were divided into four groups: non- overweight (NOW)/obese without GDM (OB- NGDM), overweight (OW)/OB- NGDM, NOW/obese with GDM (OB- GDM) and OW/OB- GDM. Linear discriminant analysis effect size (LEfSe) analysis, Spearman’s correlation analysis and t- test were performed to estimate the association amongst microbiota, pre- pregnancy BMI and oral glucose tolerance test (OGTT) glucose levels.

**Results.** *Blautia*, *Anaerostipes*, *Synergistes* (*P*<0.001) and *Christensenellaceae_R_7_group* (*P*=0.007) were significantly different between NOW/OB-GDM and OW/OB-GDM groups after adjusting for age. *Odoribacter*, *Anaerostipes*, *Monoglobus*, *Romboutsia*, *Oscillospiraceae__UCG-003*, *Blautia* and *Dialister* were significantly correlated with both OGTT 1 h (*P*<0.001) and 2 h (*P*<0.05) blood glucose levels, whilst *Lactobacillus* (*P*<0.001) were significantly correlated with OGTT 2 h blood glucose levels. *Synergistes* (*P*<0.001) were significantly correlated with OGTT fasting glucose levels, and *Megasphaera* and *Odoribacter* (*P*<0.05) were significantly correlated with pre-pregnancy BMI.

**Conclusions.** GDM and OB/OW women was experiencing microbiota dysbiosis, especially the microbial communities related to glucose metabolism.

## Introduction

Gestational diabetes mellitus (GDM) is one of the most common complications of pregnancy. A large number of studies have shown that GDM has both short-term and long-term effects on the health of mothers and offspring [1]. One in every seven live births worldwide is affected by GDM [2]. Women with GDM have an increased risk of gestational hypertension, preeclampsia, caesarean section, preterm delivery, neonatal hypoglycaemia, macrosomia and low birth weight [3,4]. Women with GDM and their offspring are at an increased risk for developing diabetes mellitus [5].

The molecular mechanisms underlying GDM remain undefined. The proposed aetiological mechanisms of obesity/overweight leading to GDM include inflammation, oxidative stress, insulin resistance and imbalance of gut microbiome [6]. Emerging epidemiological studies have found associations of overweight/obesity and excessive gestational weight gain, which are considered potential risk factors for GDM [7,9]. Studies have shown that overweight and obesity can increase the levels of inflammatory factors, such as C-reactive protein, TNF-*α* and IL-6, and decrease insulin sensitivity, thereby increasing the risk of GDM [10,11].

It is generally accepted that human gut microbiota is essential in determining the host’s health. Previous studies have found that the gut microbiota has complex relationships and correlations with obesity and the pathogenesis of GDM [12,13]. However, the results are inconsistent. A mice study revealed that, in comparison to mice of a normal weight, *Firmicutes* populations were more numerous in obese mice [14]. A study from Denmark showed that higher amounts of *Faecalibacterium* and *Anaerotruncus* and lower concentrations of *Clostridium* and *Veillonella* were observed in GDM women compared to normoglycaemic pregnant women [15]. Cui *et al*. [16] found that the abundance of *Bacteroidetes* of the GDM group was more dominant than those of the healthy group .

Several metagenomic studies have shown that gut microbiota related to carbohydrate metabolism and insulin signalling pathways are enriched in pregnant women with GDM [17]. These results indicate that there may be a link between gut microbiota and GDM and obesity. However, to the best of our knowledge, previous studies have not reported the associations between gut microbiota and the risk of GDM amongst overweight/obese women, whilst the interaction between gut microbiota dysbiosis and overweight/obesity in pregnant women with GDM remains unknown. Therefore, based on a prospective cohort study, we explored the composition of gut microbiota in overweight/obese pregnant women and its association with GDM.

## Methods

### Study sites and participants

The participants were recruited from the Pregnancy Metabolic Disease and Adverse Pregnancy Outcome (PMDAPO) study, which is an ongoing large prospective cohort study of pregnant women at Guangzhou Women and Children’s Medical Centre (GWCMC), China. The details of the cohort have been elaborated elsewhere [18]. From September 2019 to December 2021, 2,133 pregnant women were selected from the PMDAPO cohort. After excluding 313 pregnant women without faecal sample or faecal sample sequences providing <10,000 reads, 1,820 pregnant women with an available faecal sample were included in data analyses involving the gut microbiota. The exclusion criteria included (1) usage of the following drugs in the previous 6 months: systemic antibiotics, corticosterone, cytokines, methotrexate or other immunotoxic drugs, hormonal contraceptives and high dose of commercial probiotics; (2) presence of risky diseases: serious cardiovascular disease, inflammatory bowel disease, irritable bowel syndrome and coeliac disease; (3) human immunodeficiency virus infection; (4) intestinal surgery within 5 years; (5) chronic diarrhoea caused by *Clostridium difficile* or an unknown agent; (6) chronic constipation; (7) unusual dietary habits such as individuals with alcoholism and strict vegetarians; and (8) conventional antibiotic treatment or probiotic supplement in the preceding 4 weeks.

### GDM and overweight/obesity assessment

GDM was diagnosed when any of the following values from the 75 g oral glucose tolerance test (OGTT) performed at 24–28 weeks’ gestation was equalled or exceeded: fasting plasma glucose of 5.1 mmol l^−1^, 1 h plasma glucose of 10.0 mmol l^−1^ or 2 h plasma glucose of 8.5 mmol l^−1^ [19]. Overweight/obesity before pregnancy was diagnosed when BMI ≥24.0 kg m^−2^ according to the guidelines for prevention and control of overweight and obesity in Chinese adults [20]. Pre-pregnancy BMI=weight before pregnancy (kg)/square of height (m). Based on BMI before pregnancy and GDM status, pregnant women were divided into four groups: non-overweight/obese without GDM (NOW/OB-NGDM), overweight/obese without GDM (OW/OB-NGDM), non-overweight/obese with GDM (NOW/OB-GDM) and overweight/obese with GDM (OW/OB-GDM).

### Clinical data collection

Data regarding sociodemographic background, pregnancy characteristics, medical history and neonatal information were obtained from the GWCMC medical record system. Detailed individual information collected from pregnant women includes age, height, pre-pregnancy weight, place of birth and blood type. Pregnancy characteristics include gravidity, parity, history of abortion, pregnancy mode, delivery mode and time of delivery. Information regarding the medical history included the history of disease, scarred uterus, infection during pregnancy and medication during pregnancy. Neonatal information included infant sex and weight.

### Faecal sample collection and 16S rRNA sequencing testing

One faecal sample was obtained from each participant before 24 weeks of gestation when the participants attended for their routine hospital visits. Three to five grams of the central part of the last faeces were picked up using a sterile spoon and were put into a sterile faeces collection cup. If the faeces were loose and contaminated by urine or leucorrhoea, they would not be collected. For each participant, three parallel samples were subpackaged and frozen at −80 °C. All personnel responsible for specimen collection and analysis were trained to follow a standardized protocol, with clearly specified procedures and the same sequencing instruments and reagents [21]. Therefore, the batch effect should be minimal.

Total bacterial DNA was extracted from the faecal samples using the MOBIO PowerSoil^®^ DNA Isolation Kit 12888–100 protocol. We amplified the V4 region of the 16S rRNA gene using the universal 515F (5′-GTGYCAGCMGCCGCGGTAA-3′) and 806R (5′-GGACTACNVGGGTWTCTAAT-3′) primers, along with barcode sequences for each sample. Illumina NovaSeq 6000 (PE150 sequencing mode) was used to perform high-throughput sequencing. The 16S rRNA amplicon sequences were processed using QIIME2 (Ver.2021.2), and all reads were truncated at the one hundred fiftieth base with a median Q score >20 to avoid sequencing errors at the end of the reads. DADA2 was used to remove possible PCR amplification and sequencing errors in high-throughput sequencing data [22,23]. The term operational taxonomic unit (OTU) was used throughout the entire study for convenience. The taxonomy of the features was identified using the classify-sklearn classification methods based on the Greengenes 13.8 database via the q2-feature-classifier plugin (https://data.qiime2.org/2021.2/common/gg-13-8-99-515-806-nb-classifier.qza).

### Statistical analysis

The clinical data were analysed using SPSS 22.0 (SPSS Inc., Chicago, IL, USA). For continuous data, normally distributed data were analysed by ANOVA and expressed as mean±sd. For count data, the chi-square test was used. All tests were two-tailed, and *P*<0.05 was considered statistically significant.

The analysis of microbial data was performed in R (4.2.1). The Wilcoxon test was used for alpha diversity (Simpson index, Shannon index, Chao1 index and Pielou index) comparison between two groups. The Kruskal–Wallis test was used for multiple-group comparisons in alpha diversity and comparing the species composition amongst the four groups. Based on Bray–Curtis distances, principal coordinate analysis (PCoA) was used to analyse the *β*-diversity amongst the four groups, and permutational multivariate analysis of variance (PERMANOVA) was used to compare the difference of *β*-diversity amongst the four groups. The difference in relative abundance of bacterial genera between different groups was compared by the Wilcoxon test. According to our previous study, the influence of gestational age on women’s gut microbiota was minimal [21]. Therefore, we used Spearman’s correlations to assess associations amongst the differential taxa, OGTT levels and pre-pregnancy BMI values.

Linear discriminant analysis effect size (LEfSe) analysis was performed in the Galaxy web platform (usegalaxy.org) to find differential taxa amongst the four groups. In the LEfSe analysis, the microbiota data from phylum to genus were used, and the Linear Discriminant Analysis (LDA) score was set at ≥3.0. Multivariate association with linear models (MaAsLin2) [24] was performed using R (4.3.0) to adjust for age. *P*-values were corrected for multiple testing using the false discovery rate. False discovery rate-adjusted *P*<0.2 was considered statistically significant in our MaAsLin2 analysis. The minimum abundance for each feature and the minimum percent of samples for which a feature was detected at minimum abundance were set to 0, the q-value threshold for significance was set to 0.2 and other parameters were set by default.

## Results

### Sociodemographic and clinical factors associated with overweight/obesity and GDM

Amongst 1,820 pregnant women, the incidence of OB/OW, GDM and OB/OW combined with GDM (OB/OW-GDM) was 11.7% (214 out of 1,820), 24.8% (452 out of 1,820) and 4.6% (84 out of 1,820), respectively. The participants were aged 18–45 years old, the age of the OB/OW-GDM group was highest (33.0±4.5) and the age of the NOB/OW-NGDM group was lowest (30.1±4.0). The pre-pregnancy BMI, parity, gravity, abortion history and pregnancy season amongst the four groups were significantly different (Table 1). Compared with NOW/OB-NGDM, the pre-pregnancy BMI and age of other groups were higher, and the OW/OB-GDM group was the highest. The gravidity of the NOB/OW-NGDM group was lowest compared to the other three groups.

**Table 1.** Demographic characteristics of participants

Variable		OB/OW-GDM(*n*=84)	NOB/OW-GDM(*n*=368)	OB/OW-NGDM(*n*=130)	NOB/OW-NGDM(*n*=1,238)	*P*-value
Age (years)	$\bar{x}$±sd	33.0±4.5**	31.8±4.1	31.5±4.1	30.1±4.0	<0.001
Age group (years)	≤29	16 (19.0)**	104 (28.3)**	47 (36.2)**	602 (48.6)	<0.001
	30–34	37 (44.0)	169 (45.9)	50 (38.5)	456 (36.8)	
	≥35	31 (36.9)	95 (25.8)	33 (25.4)	180 (14.5)	
Pre-pregnancy BMI (kg m^−2^)	$\bar{x}$±sd	26.4±1.9**	20.4±1.9**	25.7±1.8**	19.9±1.9	<0.001
	<18.5	0**	302 (82.1)	0**	941 (76.0)	<0.001
	18.5–23.9	0	66 (17.9)	0	297 (24.0)	
	≥24.0	84 (100)	0	130 (100)	0	
Gravidity (times)	1	22 (26.2)**	138 (37.5)	30 (23.6)**	493 (40.8)	0.001
	23	30 (35.7)32 (38.1)	139 (37.8)91 (24.7)	55 (43.3)42 (33.1)	413 (34.2)302 (25.0)	
Parity (times)	0	40 (47.6)	199 (54.1)	46 (36.2)**	663 (55.2)	0.001
	1	40 (47.6)	164 (44.6)	71 (55.9)	487 (40.5)	
	2	4 (4.8)	5 (1.4)	10 (7.9)	51 (4.2)	
Pregnancy season	Spring	37 (44.0)	171 (46.5)**	52 (40.0)	429 (34.7)	0.006
	Summer	2 (2.4)	17 (4.6)	8 (6.2)	100 (8.1)	
	Autumn	12 (14.3)	52 (14.1)	21 (16.2)	195 (15.8)	
	Winter	33 (39.3)	128 (34.8)	49 (37.7)	511 (41.1)	
Abortion history	No	41 (48.8)**	228 (62.3)	77 (61.6)	800 (67.3)	0.002
	Yes	43 (51.2)	138 (37.7)	48 (38.4)	388 (32.7)	

*P*-value was calculated to compare differences amongst the four groups; **shows the significant result in post hoc multiple comparisons between NOB/OW-NGDM and the other group. **P<0.01.

### Differences of bacterial diversity

**Fig. 1.**
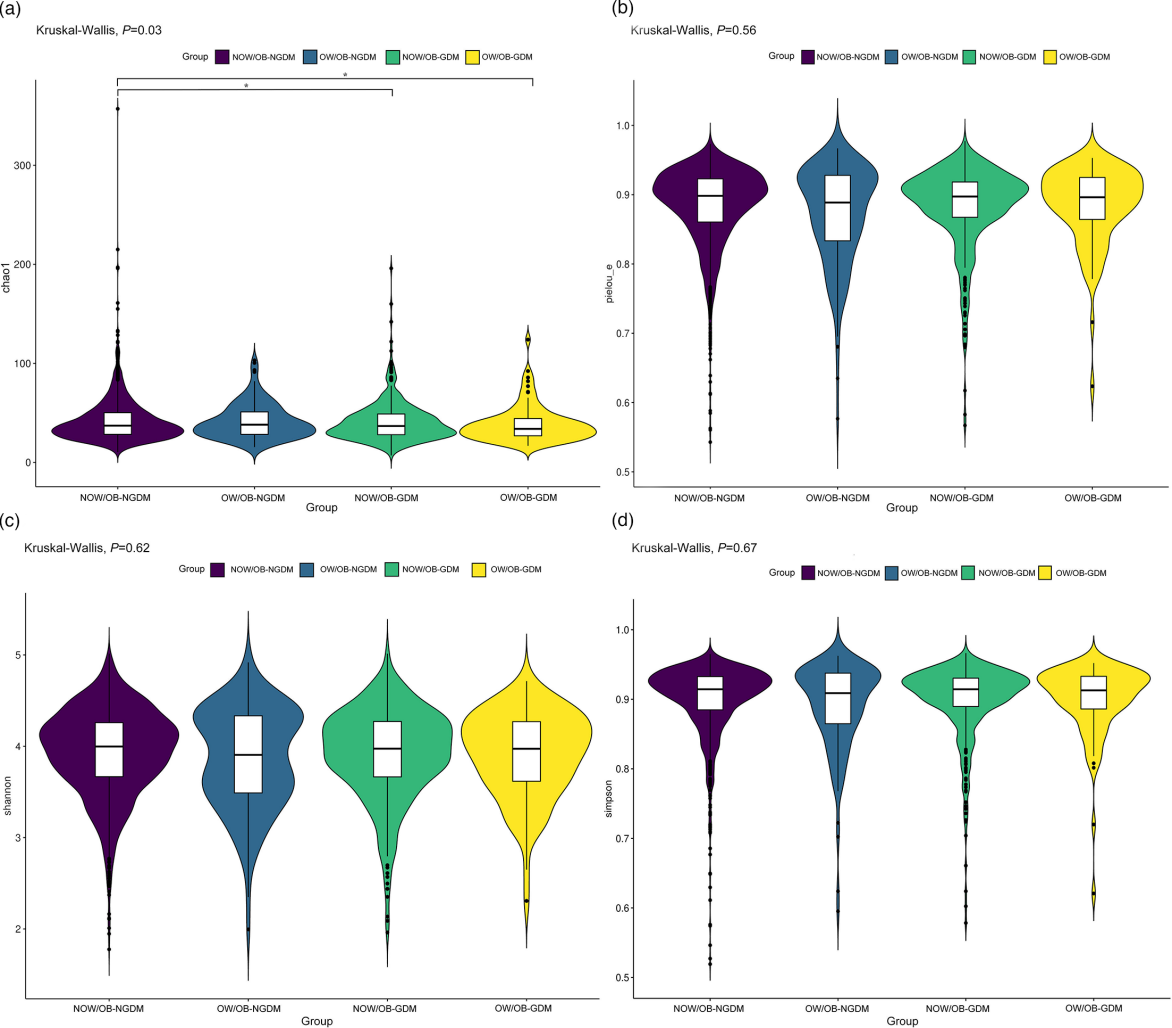
Alpha-diversity index amongst pregnant women with GDM and OW/OB. (**a**) Chao1 indices evaluate the number of OTUs in the sample, (**b**) Pielou indices evaluate the species evenness, (**c**) Shannon indices assess the richness and evenness of the species composition and (**d**) Simpson indices assess the species diversity. *P<0.05.

**Fig. 2.**
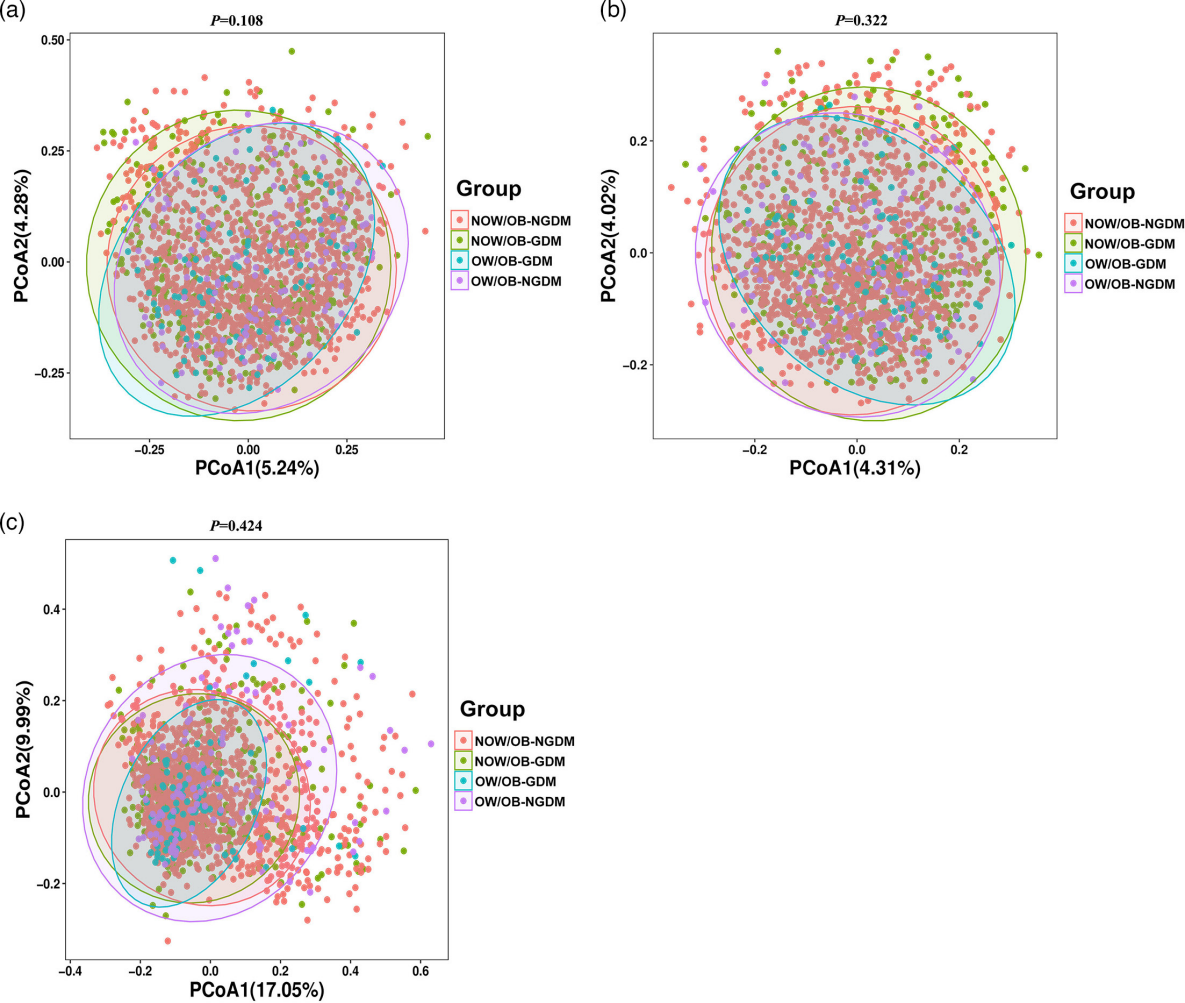
Beta diversity index between the pregnant women with GDM and OW/OB. PCoA plots of the faecal microbiome based on Bray–Curtis distances (**a**), Jaccard distances (**b**) and weighted UniFrac distances (**c**). *P*-values were obtained by two-sided PERMANOVA.

The faecal microbial *α*-diversity index Chao1 indices amongst the four groups were significantly different (*P*=0.035). Compared with the NOW/OB-NGDM group, the Chao1 indices showed that microbial alpha-diversity was significantly decreased in the pregnant women with OW/OB-GDM and NOW/OB-GDM. There were no significant differences amongst the four groups in other microbial *α*-diversity indices including the Simpson, Shannon and Pielou_e indices (Fig. 1). PCoA was used to analyse the similarity of gut microbial community structure between the four groups. Based on Bray–Curtis distances, Jaccard distances and weighted UniFrac distances, the results showed that PCoA1 and PCoA2 contributed 5.24 and 4.28%, 4.31 and 4.02% and 17.05 and 9.99%, respectively, to the difference of intestinal flora amongst the four groups (Fig. 2a–c). PERMANOVA analysis showed that there was no significant difference in intestinal flora *β*-diversity amongst the four groups (Tables S1–S3, available in the online Supplementary Material).

### Phylogenetic profiles of faecal microbial communities and correlation between differential taxa

**Fig. 3.**
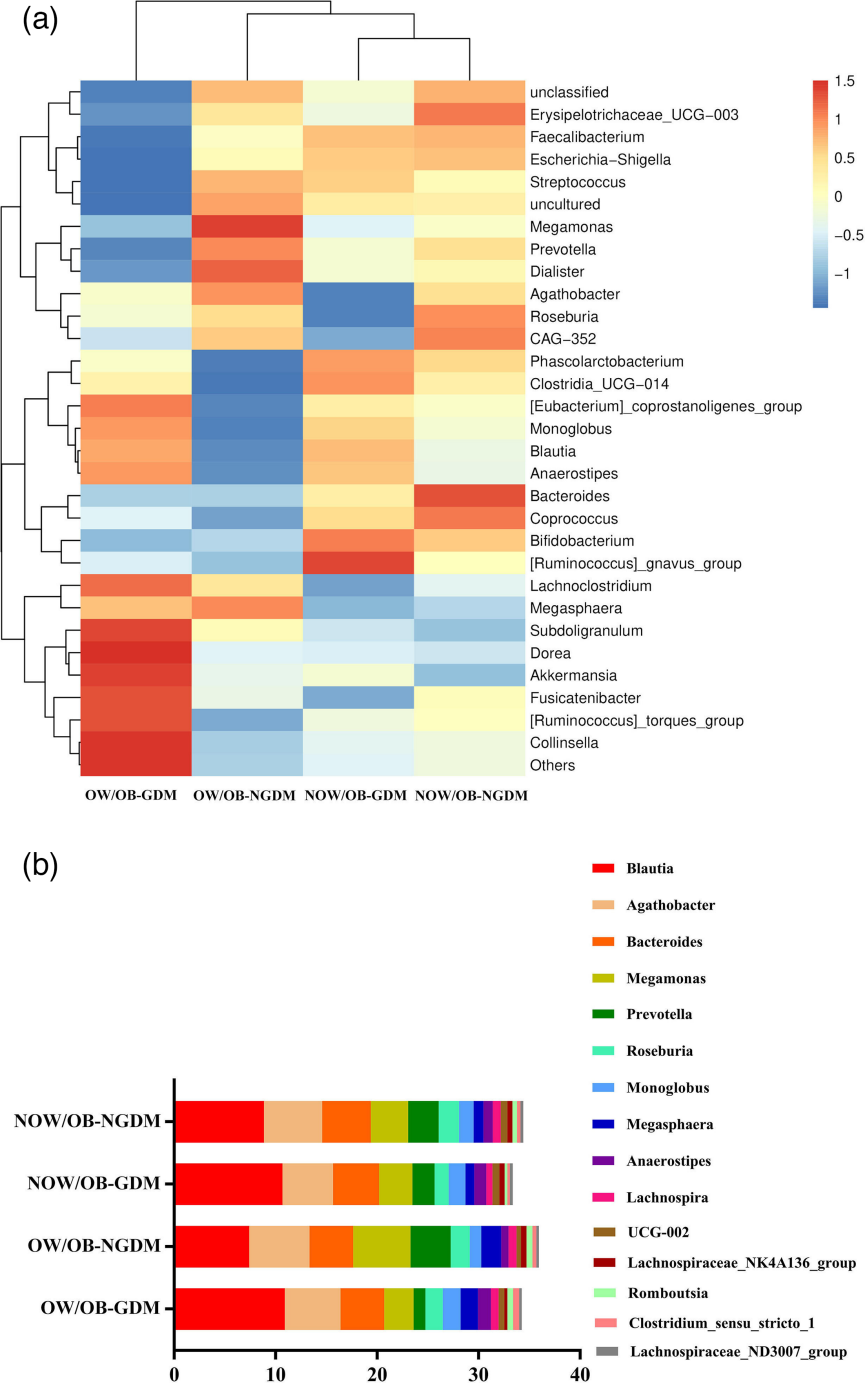
Composition and difference of bacterial taxa amongst OW/OB-GDM, OW/OB-NGDM, NOW/OB-GDM and NOW/OB-NGDM groups. (**a**) Heat map demonstrated the differential comparisons of bacterial taxa amongst the four groups. (**b**) Stacked bar plot of the top 15 significantly different bacterial abundances amongst the four groups.

Amongst all groups, *Faecalibacterium* and *Blautia* played a dominant role. Compared with pregnant women with NOW/OB-NGDM and OW/OB-NGDM, the relative abundances and respective corresponding proportions of *Blautia* were higher in pregnant women with NOW/OB-GDM and OW/OB-GDM, accounting for 10.67 and 10.91%, respectively (Fig. 3a and Table S4). Fig. 3(b) and Table S5 are the stacked bar plots of the top 15 significantly different bacterial abundances amongst the four groups. *Blautia*, *Monoglobus* and *Anaerostipes* were up-regulated in GDM women, whilst *Megasphaera*, *Romboutsia* and *Clostridium_sensu_stricto_1* were up-regulated in OW/OB women.

**Fig. 4.**
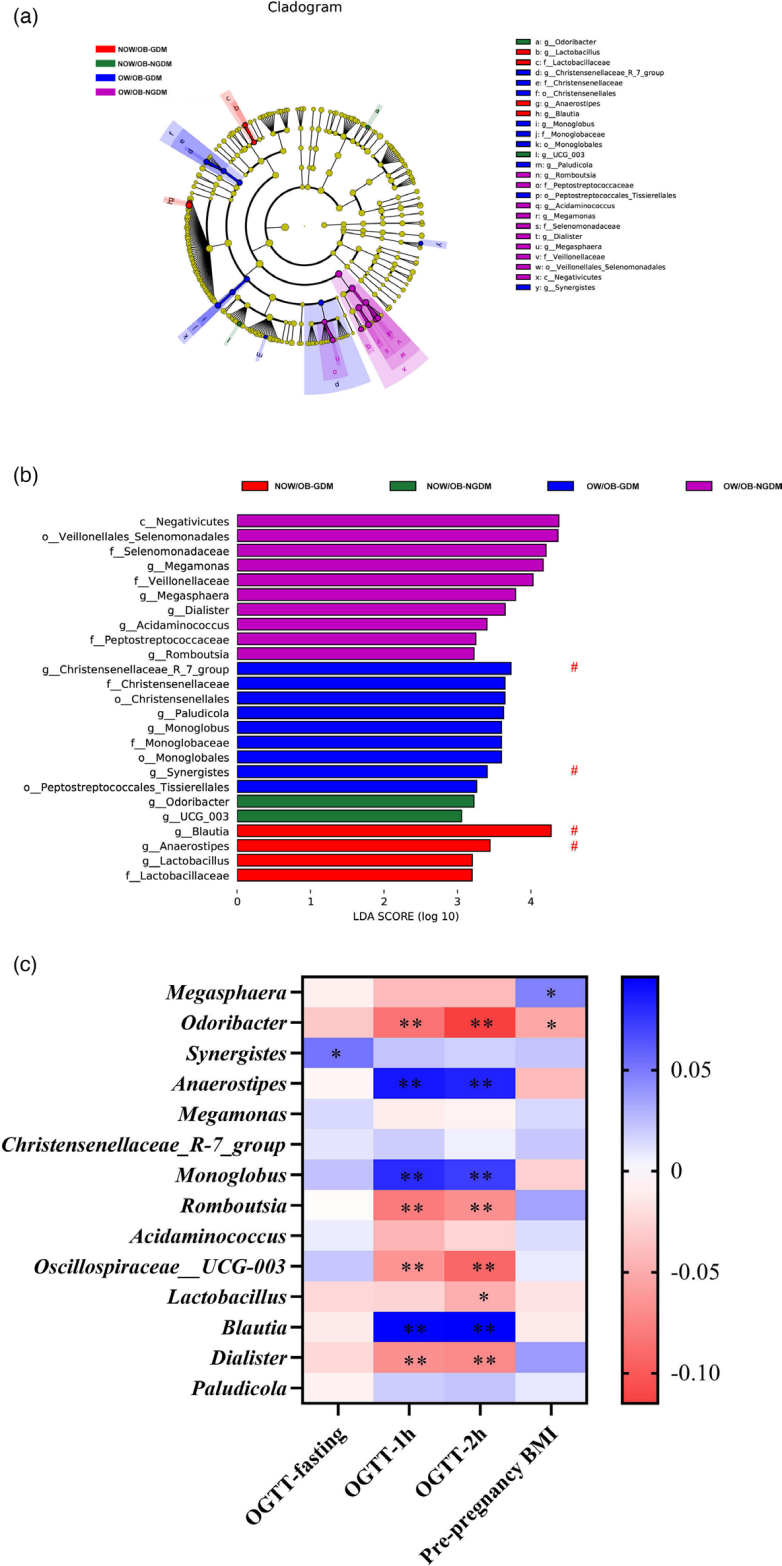
The shift of gut microbiota in OW/OB-GDM, OW/OB-NGDM, NOW/OB-GDM and NOW/OB-NGDM groups. (**a**) Cladogram and (**b**) scores of differential taxa showing the most differentially abundant taxa identified by LEfSe. Purple indicates clades enriched in the OW/OB-NGDM group, blue indicates clades enriched in the OW/OB-GDM group, green indicates clades enriched in the NOW/OB-NGDM group and red indicates clades enriched in the NOW/OB-GDM group. Only genera meeting an LDA score threshold >3 are shown. # means significant genus after adjusting for age using MaAsLin2. (**c**) Heat map of the correlation amongst the different taxa, OGTT and pre-pregnancy BMI. *P<0.05; **P<0.001. All microbiota data (*n*=1,820) were used.

To further verify the differentially abundant taxa in the pregnant women with OW/OB-GDM, based on the 16S rRNA gene sequencing, the LDA coupled with effect size analysis (LEfSe) algorithms was performed on faecal microbiota composition amongst the pregnant women with OW/OB-GDM, NOW/OB-GDM, OW/OB-NGDM and NOW/OB-NGDM, respectively. The results of LEfSe revealed that there was a total of 25 different bacteria amongst the pregnant women in four groups. At the genus level, *Megasphaera*, *Megamonas*, *Romboutsia*, *Acidaminococcus* and *Dialister* were enriched in OW/OB-NGDM pregnant women; *Synergistes*, *Christensenellaceae_R_7_group*, *Monoglobus* and *Paludicola* were enriched in OW/OB-GDM pregnant women; *Odoribacter* and *Oscillospiraceae* _UCG_003 were enriched in NOW/OB-NGDM pregnant women; and *Anaerostipes*, *Lactobacillus* and *Blautia* were enriched in NOW/OB-GDM pregnant women. After adjusting for age using the multivariate association with the linear model (MaAsLin2) method, *Blautia*, *Anaerostipes*, *Christensenellaceae_R_7_group* and *Synergistes* were still significantly different between NOW/OB-GDM and OW/OB-GDM groups (Fig. 4a, b).

The rank correlation amongst the differential taxa based on all microbiota data (*n*=1820), OGTT and pre-pregnancy BMI showed that *Odoribacter*, *Anaerostipes*, *Monoglobus*, *Romboutsia*, *Oscillospiraceae*_*UCG-003*, *Blautia* and *Dialister* were significantly correlated with both 1 h glucose of OGTT (*P*<0.001) and 2 h glucose of OGTT (*P*<0.05), whilst *Lactobacillus* (*P*<0.001) was significantly correlated with 2 h glucose of OGTT. *Synergistes* (*P*<0.001) were significantly correlated with fasting glucose of OGTT, and *Megasphaera* and *Odoribacter* (*P*<0.05) were significantly correlated with pre-pregnancy BMI (Fig. 4c and Table S6).

**Fig. 5.**
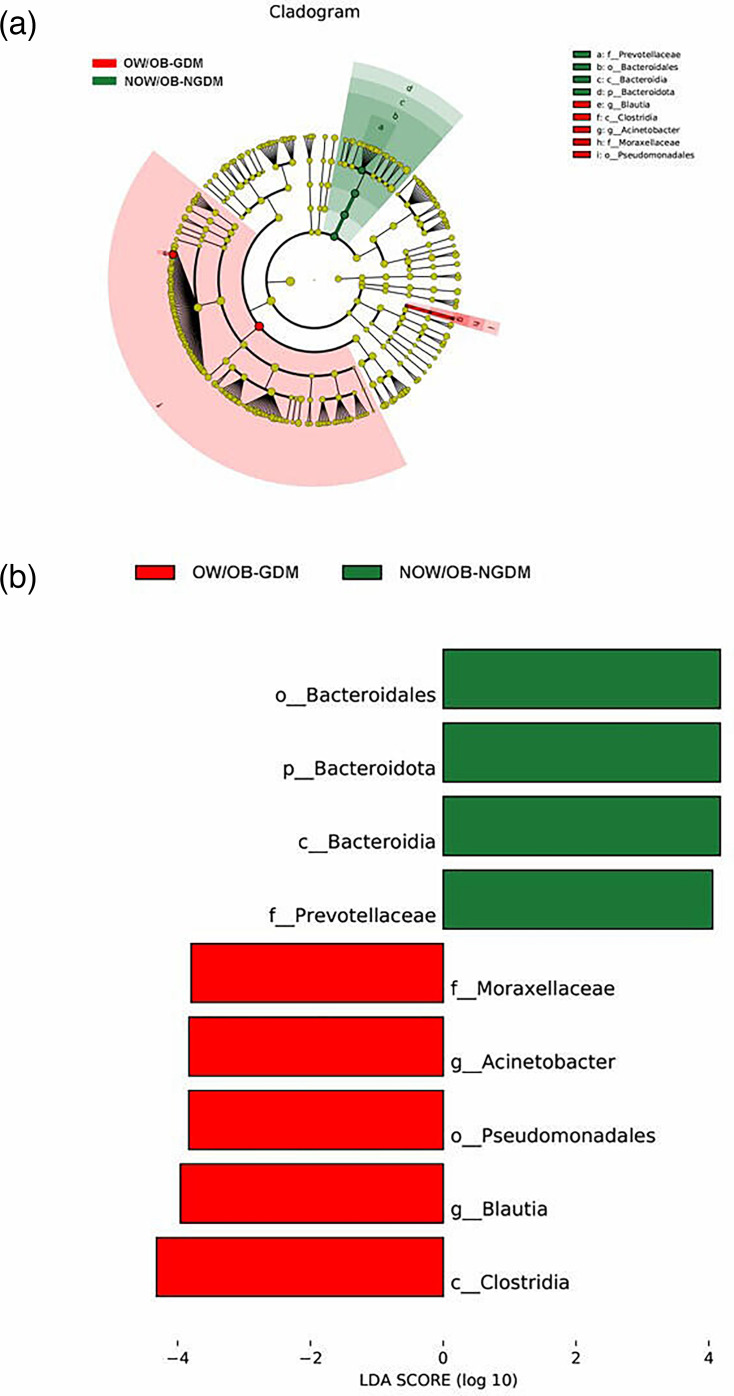
Pairwise LEfSe analysis of gut microbiota in OW/OB-GDM and NOW/OB-NGDM groups. (**a**) Cladogram and (**b**) scores of differential taxa showing the most differentially abundant taxa identified by LEfSe. Red indicates clades enriched in the OW/OB-GDM group, and green indicates clades enriched in the NOW/OB-NGDM group. Only genera meeting an LDA score threshold >3 are shown.

**Fig. 6.**
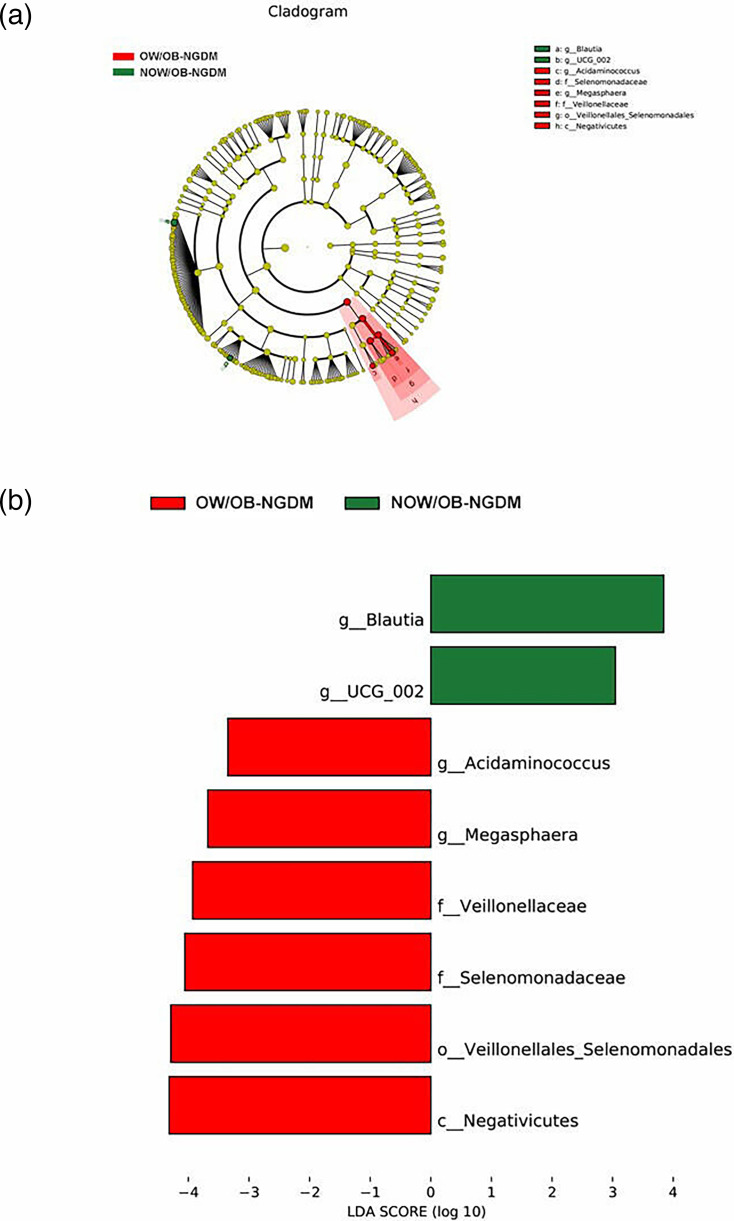
Pairwise LEfSe analysis of gut microbiota in OW/OB-NGDM and NOW/OB-NGDM groups. (**a**) Cladogram and (**b**) scores of differential taxa showing the most differentially abundant taxa identified by LEfSe. Red indicates clades enriched in the OW/OB-NGDM group, and green indicates clades enriched in the NOW/OB-NGDM group. Only genera meeting an LDA score threshold >3 are shown.

**Fig. 7.**
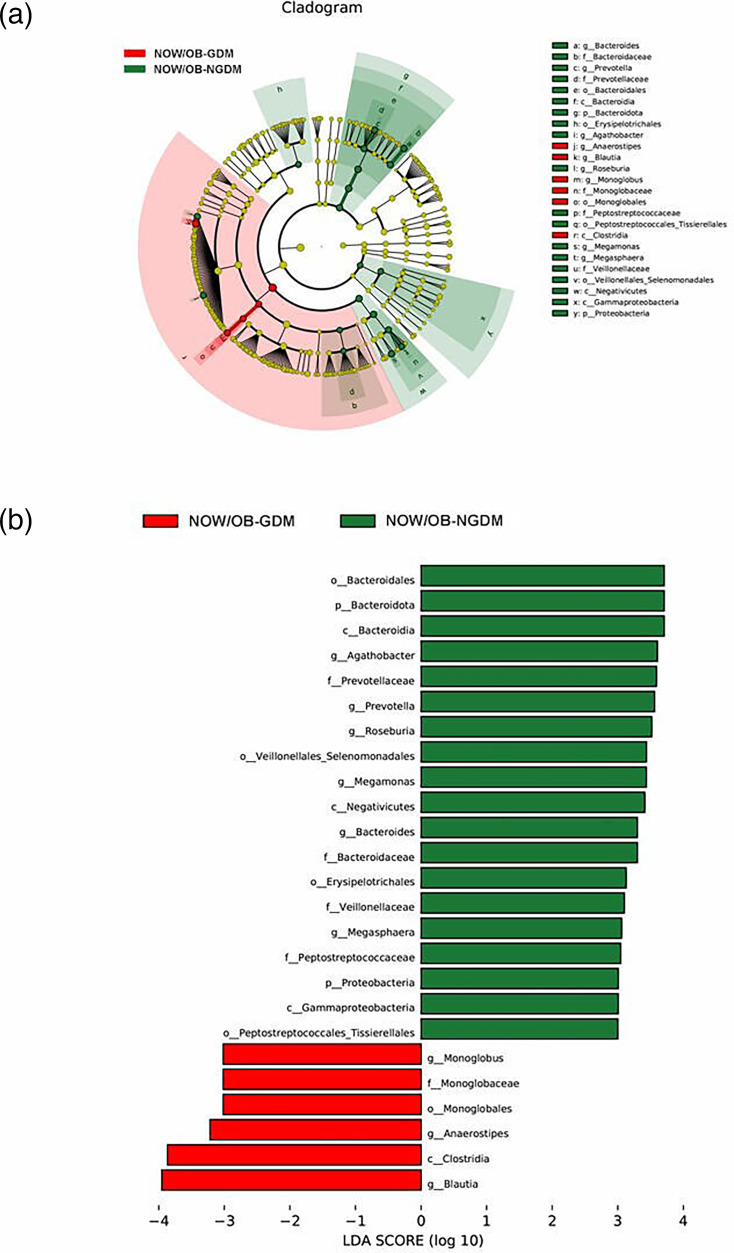
Pairwise LEfSe analysis of gut microbiota in NOW/OB-GDM and NOW/OB-NGDM groups. (**a**) Cladogram and (**b**) scores of differential taxa showing the most differentially abundant taxa identified by LEfSe. Red indicates clades enriched in the NOW/OB-GDM group, and green indicates clades enriched in the NOW/OB-NGDM group. Only genera meeting an LDA score threshold >3 are shown.

To further explore the marker bacteria between GDM, OW/OB women and healthy women (NOW/OB-NGDM), we performed a pairwise LEfSe analysis. The results showed that at the genus level, compared to the NOW/OB-NGDM women, *Acinetobacter* and *Blautia* were enriched in OW/OB-GDM women (Fig. 5a, b), *Acidaminococcus* and *Megasphaera* were enriched in OW/OB-NGDM women (Fig. 6a, b) and *Monoglobus, Anaerostipes* and *Blautia* were enriched in NOW/OB-GDM women (Fig. 7a, b). The above results suggested that anaerobic bacteria and *Bacillus* might be markers of GDM, and *Acidaminococcus* and *Megasphaera* were considered to be the marker bacteria of OW/OB.

## Discussion

In this study, we found that GDM and OW/OB women were characterized by *Bacteroidota*, *Synergistes* and *Firmicutes*. After adjusting for age, *Blautia*, *Anaerostipes*, *Christensenellaceae_R_7_group* and *Synergistes* were still significantly different between NOW/OB-GDM and OW/OB-GDM.

Compared with NOW/OB-NGDM, the Chao1 indices showed that microbial alpha-diversity was significantly decreased in the pregnant women with OW/OB-GDM and NOW/OB-GDM. Several studies showed that there was no significant difference in the alpha diversity between GDM and non-GDM, OW/OB and NOW/OB pregnant women [25,26]. Whilst the results of Abdullah *et al*. [27] showed a trend of relatively lower diversity indices (Ace, Chao, Shannon and Simpson) in the gut microbiota profiles of GDM than in non-GDM pregnant women, Song and Liu [28] also revealed that Chao1 indices in women with excessive pregnancy weight gain significantly decreased compared to women with normal pregnancy weight gain. No significant difference in *β*-diversity was observed amongst the four groups in our study; this was consistent with the previous studies [29,30]. However, a cohort study observed a significantly different *β*-diversity amongst women with GDM, obesity and normal weight [31].

Although the pathogenesis of GDM remains unclear, increasing studies indicate that GDM is closely associated with overweight/obesity and microbe dysbiosis. A study of obese mice reported that obese mice had a 50% reduction in the abundance of *Bacteroidetes* and a proportional increase in *Firmicutes* [32]. Other studies also revealed that an increased abundance of *Firmicutes* reduced the richness of *Bacteroidota*, *Roseburia* and *Dialister* in GDM women compared with normoglycaemic women [33,34]. Similarly, our results also found that *Bacteroidota* were downregulated in OW/OB and GDM women, whilst some *Firmicutes* (such as *Blautia*) were upregulated in GDM women and downregulated in OW/OB-NGDM women. Besides, a longitudinal study revealed that *Bacteroidota* were associated with GDM, and *Anaerostipes* were associated with impaired glucose tolerance [35]. Our results also showed that *Anaerostipes* were enriched in NOW/OB-GDM women. A decrease of *Lactobacillus* was also shown in GDM women compared with pregnant women with normoglycaemia [36]. This was consistent with our findings showing that *Lactobacillus* was significantly negatively correlated with 2 h glucose of OGTT.

Emerging evidence indicates that the underlying pathology of GDM and overweight/obesity may be the alteration of butyrate-producing bacteria. A metagenome-wide association study showed that type 2 diabetes was characterized by a moderate degree of gut microbial dysbiosis and a decrease in the abundance of some universal butyrate-producing bacteria [37]. Butyrate is a metabolite of short-chain fatty acids (SCFAs), which can activate intestinal gluconeogenesis via a gut-brain neural circuit, thereby promoting metabolic benefits on body weight and glucose control [38]. Therefore, the butyrate-producing bacteria contribute to the host energy acquisition and metabolic regulation, thereby influencing the development of metabolic disorders such as obesity and diabetes [39]. A study also found that butyrate improves insulin sensitivity and increases energy expenditure in mice [40]. After adjusting for age, *Blautia* and *Anaerostipes*, which belong to butyrate-producing bacteria, were still significantly enriched in NOW/OB-GDM women. They may be the pathogenic targets of GDM. Furthermore, *Christensenellaceae_R_7_group* and *Synergistes* were also enriched in OW/OB-GDM in our study. *Christensenellaceae* were reported to be associated with increased fasting plasma glucose and hosting BMI [16,41]. To our best knowledge, the relationship between *Synergistes* and GDM or overweight/obesity has not been reported, which may be a potential candidate to further investigate the underlying mechanism of related diseases. Additionally, our pairwise LEfSe results revealed that *Acidaminococcus* and *Megasphaera* might be the marker bacteria associated with OW/OB, and this is consistent with the previous study [42].

*Blautia* and *Anaerostipes*, which were different bacteria between NOW/OB-GDM and OW/OB-GDM pregnant women after adjusting for age, were significantly positively correlated with 1 and 2 h glucose of OGTT, and *Synergistes* were significantly positively correlated with fasting glucose. Similarly, Crusell *et al*. [15] also found that *Blautia* were positively associated with 2 h glucose of OGTT, and Li *et al*. [43] reported a significant association between *Synergistes* and 1 h glucose of OGTT. It suggests that gut microbiota might potentially contribute to GDM pathogenesis by disturbing the host’s carbohydrate metabolism.

To the best of our knowledge, this is the first study that provided epidemiological evidence of gut microbiota alterations in the associations between overweight/obese and GDM. This study has benefited from several methodological strengths. First, to obtain more accurate differential microbiota, we performed the LEfSe analysis with MaAsLin2 correction. Secondly, faecal specimens were collected before 24 weeks of gestation. The prospective cohort study design allowed us to analyse the causality between overweight/obesity and GDM. The limitations of this study cannot be ignored. Due to a lack of information on diet and exercise, residual confounding factors may have influenced the study results. Because the metabolites (such as SCFAs) in pregnant women were not obtained, the mechanisms involved could not be further explored. Only one faecal specimen was collected before 24 weeks of gestation; thus, the difference in gut microbiota amongst different gestational ages might be confused. Nevertheless, our previous study demonstrated that changes in gestational age have a limited effect on women’s gut microbiota [21].

## Conclusions

Our findings demonstrate that GDM and OB/OW women were experiencing microbiota dysbiosis, especially the microbial communities related to glucose metabolism. This study may inform the importance of weight-gain management during pregnancy to significantly reduce the risk of GDM.

## Supplementary material

10.1099/jmm.0.002010 Uncited Supplementary Material 1.

## Abbreviations

GDM: gestational diabetes mellitus
GWCMC: Guangzhou Women and Children’s Medical Centre
LDA: Linear Discriminant Analysis
LEfSe: linear discriminant analysis effect size
MaAsLin2: Multivariate association with linear models
NOW: non-overweight
OB-GDM: obese with GDM
OB-NGDM: obese without GDM
OGTT: oral glucose tolerance test
OTU: operational taxonomic unit
OW: overweight
PCoA: principal coordinate analysis
PERMANOVA: permutational multivariate analysis of variance
PMDAPO: pregnancy metabolic disease and adverse pregnancy outcome
SCFAs: short-chain fatty acids
