# Supplementary material for: Association of gut microbiota with overweight/obesity combined with gestational diabetes mellitus
Source: J Med Microbiol. 2025 May 14;74(5):002010. doi: 10.1099/jmm.0.002010 (PMC12282309; doi:10.1099/jmm.0.002010)
Supplement: Uncited Supplementary Material 1. [file jmm-74-02010-s001.pdf]

Table S1 PERMANOVA analysis based on Bray-Curtis distance of microbial β-diversity

	Df	Sum of squares	R^2	F	*P*-value
Group	3	1.77	0.003	1.69	0.108
Residual	1816	634.77	0.997		
Total	1819	636.55	1.000		

Table S2 PERMANOVA analysis based on Jaccard distances of microbial β-diversity

	Df	Sum of squares	R^2	F	*P*-value
Group	3	1.75	0.002	1.54	0.322
Residual	1816	687.40	0.998		
Total	1819	689.15	1.000		

Table S3 PERMANOVA analysis based on weighted UniFrac distances of microbial β-diversity

	Df	Sum of squares	R^2	F	*P*-value
Group	3	0.74	0.004	2.43	0.424
Residual	1816	182.95	0.996		
Total	1819	183.68	1.000		

Table S4 Relative abundance of top 30 gut microbial communities at the genus level

Genus	NOW/OB-GDM	NOW/OB-NGDM	OW/OB-GDM	OW/OB-NGDM	*P*-value
Faecalibacterium	10.78	10.83	9.22	10.24	0.39
Blautia	10.67	8.85	10.91	7.39	0.001
Subdoligranulum	7.56	7.27	9.58	8.23	0.36
Agathobacter	4.97	5.72	5.47	5.93	0.02
Bacteroides	4.55	4.80	4.30	4.31	0.001
Streptococcus	4.05	3.86	3.35	4.11	0.91
Dorea	3.73	3.71	4.19	3.74	0.68
Megamonas	3.28	3.68	2.88	5.65	0.01
Bifidobacterium	4.09	3.67	2.48	2.63	0.28

Tannerellaceae__ unclassified	2.81	3.04	2.56	3.02	0.01
Prevotella	2.18	3.01	1.19	3.97	0.02
Fusicatenibacter	2.59	2.73	2.87	2.68	0.36
Ruminococcus_torques_group	2.55	2.61	2.95	2.35	0.45
Phascolarctobacterium	1.97	1.93	1.86	1.72	0.75
Roseburia	1.39	2.01	1.69	1.87	0.001
Collinsella	1.50	1.57	2.52	1.33	0.82
Monoglobus	1.65	1.43	1.77	1.13	0.001
Ruminococcus_gnavus_group	1.62	1.30	1.20	1.12	0.97
Eubacterium_coprostanoligenes_group	1.27	1.24	1.36	1.12	0.39
Clostridia_UCG-014	1.44	1.18	1.18	0.75	0.37
Coprococcus	1.20	1.21	1.17	1.16	0.59
Lachnoclostridium	1.04	1.08	1.16	1.12	0.24
Megasphaera	0.87	0.97	1.71	1.97	0.001
Akkermansia	1.08	0.93	1.47	1.04	0.51
Anaerostipes	1.19	0.92	1.28	0.72	0.001
Ruminococcaceae_ CAG-352	0.75	1.02	0.80	0.96	0.45
Escherichia-Shigella	0.95	0.97	0.63	0.86	0.19
Dialister	0.84	0.94	0.54	1.47	0.001
Erysipelotrichaceae_UCG-003	0.89	0.96	0.84	0.92	0.42
Atopobiaceae__ uncultured	0.90	0.90	0.77	0.95	0.48
Others	15.62	15.67	16.09	15.54	0.39

Table S5 Top 15 significantly different bacteria mean abundance among the four groups

Genus	NOW/OB-GDM	NOW/OB-NGDM	OW/OB-GDM	OW/OB-NGDM	*P*-value
Blautia	10.67	8.85	10.91	7.39	0.001
Agathobacter	4.97	5.72	5.47	5.93	0.01
Bacteroides	4.55	4.80	4.31	4.31	0.001
Megamonas	3.28	3.68	2.88	5.65	0.01
Prevotella	2.18	3.01	1.19	3.97	0.01
Roseburia	1.39	2.01	1.69	1.87	0.001
Monoglobus	1.65	1.43	1.77	1.13	0.001

Megasphaera	0.87	0.97	1.71	1.97	0.001
Anaerostipes	1.19	0.92	1.28	0.72	0.001
Lachnospira	0.60	0.78	0.75	0.77	0.01
UCG-002	0.69	0.66	0.57	0.44	0.01
Lachnospiraceae_NK4A136_group	0.51	0.49	0.30	0.55	0.01
Romboutsia	0.28	0.45	0.56	0.59	0.001
Clostridium_sensu_stricto_1	0.24	0.34	0.62	0.38	0.001
Lachnospiraceae_ND3007_group	0.29	0.28	0.25	0.25	0.04

Table S6 Correlation analysis among OGTT glucose levels and the bacterial taxa (*P<0.05 **P<0.001)

Bacterial taxa	Fasting glucose of OGTT	1-h glucose of OGTT	2-h glucose of OGTT	Pre-pregnancy BMI
g__Megasphaera	-0.01	-0.041	-0.042	0.047*
g__Odoribacter	-0.034	-0.084**	-0.115**	-0.053*
g__Synergistes	0.052*	0.022	0.017	0.022
g__Anaerostipes	-0.006	0.087**	0.083**	-0.041
g__Megamonas	0.014	-0.012	-0.008	0.015
Christensenellaceae_R-7_group	0.01	0.019	0.006	0.021
g__Christensenellaceae_R-7_group	0.023	0.080**	0.073**	-0.028
g__Monoglobus	-0.002	-0.079**	-0.068**	0.035
g__Romboutsia	0.007	-0.045	-0.026	0.013
g__Acidaminococcus	0.021	-0.065**	-0.092**	0.008
g__ Oscillospiraceae__UCG-003	-0.024	-0.027	-0.049*	-0.018
g__Lactobacillus	-0.013	0.095**	0.096**	-0.013
g__Blautia	-0.023	-0.067**	-0.071**	0.037
g__Dialister	-0.009	0.019	0.022	0.009
g__Paludicola	-0.01	-0.041	-0.042	0.047*
